# Suitability of endogenous reference genes for gene expression studies with human intraocular endothelial cells

**DOI:** 10.1186/1756-0500-6-46

**Published:** 2013-02-04

**Authors:** Ruoxin Wei, Elizabeth Anne Stewart, Winfried M Amoaku

**Affiliations:** 1 University of Nottingham, Division of Ophthalmology and Visual Sciences, B Floor, Eye and ENT Building, Queen’s Medical Centre, Nottingham, NG7 2UH, UK

**Keywords:** Choroid, Retina, Endothelial cells, Housekeeping gene, Endogenous control, Polymerase chain reaction

## Abstract

**Background:**

The use of quantitative real-time reverse transcription polymerase chain reaction (qRT-PCR) has become widely applied as a method to measure transcript abundance. In order to be reflective of biological processes during health and disease this method is dependent on normalisation of data against stable endogenous controls. However, these genes can vary in their stability in different cell types. The importance of reference gene validation for a particular cell type is now well recognised and is an important step in any gene expression study.

**Results:**

Cultured primary human choroidal and retinal endothelial cells were treated with the immunostimulant polyinosinic: polycytidylic acid or untreated. qRT-PCR was used to quantify the expression levels of 10 commonly used endogenous control genes, *TBP*, *HPRT1*, *GAPDH*, *GUSB*, *PPIA*, *RPLP0*, *B2M*, *18S rRNA*, *PGK1* and *ACTB*. Three different mathematical algorithms, GeNorm, NormFinder, and BestKeeper were used to analyse gene stability to give the most representative validation. In choroidal endothelial cells the most stable genes were ranked as *HPRT1* and *GUSB* by GeNorm and NormFinder and *HPRT1* and *PPIA* by BestKeeper. In retinal endothelial cells the most stable genes ranked were *TBP* and *PGK1* by GeNorm and NormFinder and *HPRT1* by BestKeeper. The least stable gene for both cell types was *18S* with all 3 algorithms.

**Conclusions:**

We have identified the most stable endogenous control genes in intraocular endothelial cells. It is suggested future qRT-PCR studies using these cells would benefit from adopting the genes identified in this study as the most appropriate endogenous control genes.

## Background

Gene expression analysis is important in the identification of new biological and disease mechanisms. Real-time reverse transcription quantitative polymerase chain reaction (qRT-PCR) is one of the most widely applied methods to measure transcript abundance. Whether the results are truly reflective of biological processes is dependent on normalisation of data against stable endogenous controls, often referred to as housekeeping genes. A housekeeping gene is a constitutively expressed gene that is expressed in all cells of an organism [1]. An ideal housekeeping gene should be expressed at the same level in different types of cells [2]. However, whilst some of the reference genes are expressed at relatively constant levels, others may vary depending on the experimental conditions, [3] and sample type and quality [4]. A number of reports indicate that no housekeeping gene can be considered to be suitable for all conditions [2,5-7]. Such variation of reference genes may lead to inaccurate measurement of expression levels of target genes and a significant alteration of results [8]. Thus, choosing an appropriate reference gene is critical in validating the quality of gene expression studies especially using qRT-PCR. A proper validation of endogenous controls has already been extensively applied to various tissues, and become an essential requirement for accurate qRT-PCR analysis [9]. To date, despite the increase in gene expression research on intraocular endothelial cells, there are no reports of validated set of reference genes for human intraocular endothelial cells, including human choroidal endothelial microvascular cells (hCEC), and human retinal endothelial microvascular cells (hREC).

Toll-like receptors (TLRs) are known to play a key role in innate immune responses, inflammation [10] and angiogenesis [11]. TLRs are expressed in many cell types, although their localisation within the cells varies, and are activated by several endogenous and exogenous substances [12]. In the eye TLRs are thought to be important in the inflammatory diseases [13,14] diabetic retinopathy [15], and age-related macular degeneration (AMD) [16-18]. Angiogenic factors and pro-inflammatory cytokines are secreted after the activation of TLR-mediated signalling pathways when stimulated by specific ligands such as double-stranded RNA (TLR3-specific) and lipopolysaccharide (TLR4-specific) [19,20]. Polyinosinic:polycytidylic acid (Poly(I:C)), an immunostimulant, is a well characterised TLR3 ligand [21]. Poly(I:C) (TLR3) stimulation of intraocular endothelial cells, therefore, provides a suitable model for investigating housekeeping gene stability in these cells under stimulated conditions.

There are several statistical programmes developed to assess the appropriateness of reference genes. Most of them use different approaches to evaluating reference stability, and have previously been reported to yield different rankings of reference genes. These include GeNorm [4], BestKeeper [22] and NormFinder [23] which are thought to determine the most stable reference genes from a set of tested genes for each tissue sample.

GeNorm, a Visual Basic Application for Microsoft Excel, relies on the principle that the expression ratio of two ideal reference genes is identical in all samples, regardless of the experimental condition or cell type [4]. GeNorm provides a measure of gene expression stability *M* for each reference gene as the average pairwise variation *V* between that individual gene and all other tested candidate genes. For every combination of two internal control genes *j* and *k*, an array *A*_*jk*_ which consist of logarithmically transformed expression ratios is calculated (m). The gene-stability measure *M*_*j*_ is calculated as the arithmetic mean of all pairwise variations *V*_*jk*_[4]*.* Genes with the most stable expression have the lowest *M* value, and the gene with the highest *M* value has the least stable expression. Stepwise exclusion of the gene with the highest *M* value allows ranking of the tested genes according to their expression stability, ending with a combination of the two most stable genes left. Pairwise variation (V) is the level of variation in average reference gene stability with the sequential addition of each reference gene to the equation (for calculation of the normalisation factor). This starts with the two most stably expressed genes, followed by the inclusion of a 3rd, 4th, 5th gene etc. A large variation means that the added gene has a significant effect and should preferably be included for calculation of a reliable normalization factor. Most publications use 0.15 as a cut-off value, below which the inclusion of an additional reference gene is not required [24]. Another Excel-based program, BestKeeper, uses repeated pair-wise correlation analysis to determine the optimal reference genes [22]. This program uses cycle threshold (Ct) values of candidate reference genes instead of relative quantities. It employs pair-wise correlation analyses to calculate the *Pearson correlation coefficient* (*r*) for each candidate reference gene pair as well as the probability of correlation significance (*p*-value). Initial estimation of the data calculated variations (standard deviation, SD and coefficient of variance, CV values) for all the candidate reference genes show the overall stability in gene expression, from the most stable expression (with the lowest variation) to the least stable one (with the highest variation). Any candidate gene with the SD value higher than 1 is considered inconsistent [22]. Furthermore, each sample is analysed as efficiency corrected intrinsic variation of x-fold, over or under expression. Over 3-fold over- or under- expression is considered to be removed [22].

A further Visual Basic Application, NormFinder, assesses all reference candidate genes by a model-based approach based on inter- and intra-group gene expression variations [23]. NormFinder analyses data on a linear scale through any quantitative method using a model-based approach [23]. The candidate genes are assigned a value as a measure of their stability. Lower values are indicative of low intra- and intergroup variations and of greater stability. NormFinder calculates the stability value for all candidate normalization genes tested in the sample set, and selects two best genes with minimal combined inter- and intra- group expression variation [23].

The aim of this study was to determine the most suitable and stable housekeeping genes for use in the study of hCEC and hREC gene expression with RT-qPCR. This was done by assessing the stability of 10 commonly used housekeeping genes using three different mathematical algorithms in both untreated and poly(I:C) stimulated hCEC and hREC.

## Results and Discussion

A number of studies on the validation of reference genes have been done for different species, and tissues. Software-based applications such as GeNorm, NormFinder, BestKeeper were used to perform statistical identification of the best reference gene from a group of candidate genes in a defined set of biological samples. Ten commonly used qPCR reference genes were investigated for the expression stability in hCEC and hREC using three software programs. Although the three programs produced similar results, the ranking of investigated genes were not identical due to the different algorithms [25]. As the three programs, GeNorm, NormFinder and BestKeeper, use different approaches to evaluating reference stability; have been previously reported to yield different rankings of reference genes [25]. The M-values obtained from GeNorm, variability measurements from NormFinder, and the coefficients of correlation from BestKeeper were used as weights in the aggregation process.

### Expression of the endogenous control genes in hCEC and hREC

**Figure 1:**
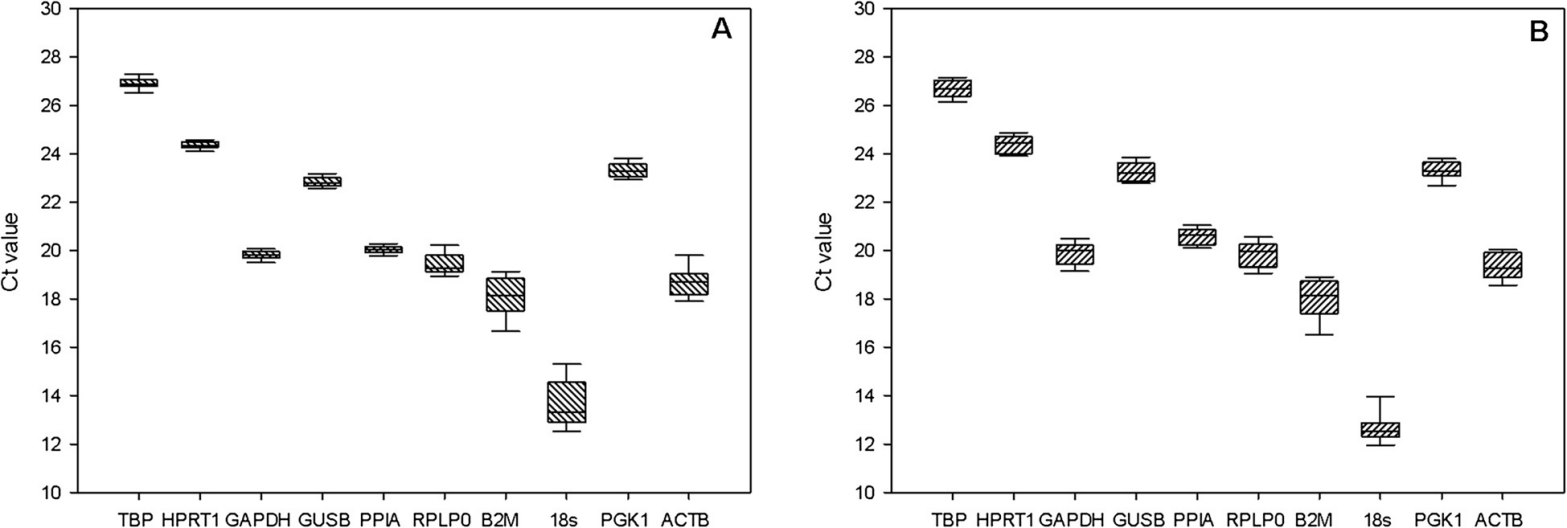
**The distribution of gene expression levels of candidate reference genes in hCEC (A) and hREC (B).** Values are given as qRT-PCR Ct values. The boxes represent the 25 to 75 percentile ranges with medians (line in the box); the whiskers illustrate the 1 to 99 percentile of the samples.

The expression of 10 commonly used reference genes, including *TBP*, *HPRT1*, *GAPDH*, *GUSB*, *PPIA*, *RPLP0*, *B2M*, *18S*, *PGK1* and *ACTB*[26] (Table 1) were measured using unstimulated and stimulated hCEC and hREC using qRT-PCR (Figure 1).

**Table 1:** Candidate reference genes for qRT-PCR

**Abbreviation**	**Gene name**	**Function**
*18S*	18S Ribosomal RNA	Ribosomal subunit
*ACTB*	β-actin	Cytoskeletal structural protein
*B2M*	β-2-microglobulin	β-chain of major histocompatibility complex class I molecules
*GAPDH*	Glyceraldehyde-3- phosphate dehydrogenase	Oxidoreductase in glycolysis and gluconeogenesis
*GUSB*	beta-glucuronidase	Degradation of determatan and keratin sulfates
*HPRT1*	Hypoxanthine phosphoribosyl transferase 1	Purine synthesis in salvage pathway
*PGK1*	Phosphoglycerate kinase 1	Glycolytic enzyme
*PPIA*	Peptidylprolyl isomerase A	Catalyzes the cis-trans isomerisation of praline, accelerates protein folding
*RPLP0*	Ribosomal large P0	Ribosomal protein, translation
*TBP*	TATA binding protein	RNA polymerase II transcription factor

### Reference gene validation in hCEC

Endogenous gene expression in 12 hCEC samples (1 unstimulated hCEC and 3 stimulated with poly(I:C) for 1h, 6h or 24h for each of 3 individual donor samples) was analysed with each of the 3 mathematical algorithms. The input data for GeNorm and NormFinder was relative quantities transformed by the comparative Ct method, while the data for BestKeeper was raw Ct values.

**Figure 2:**
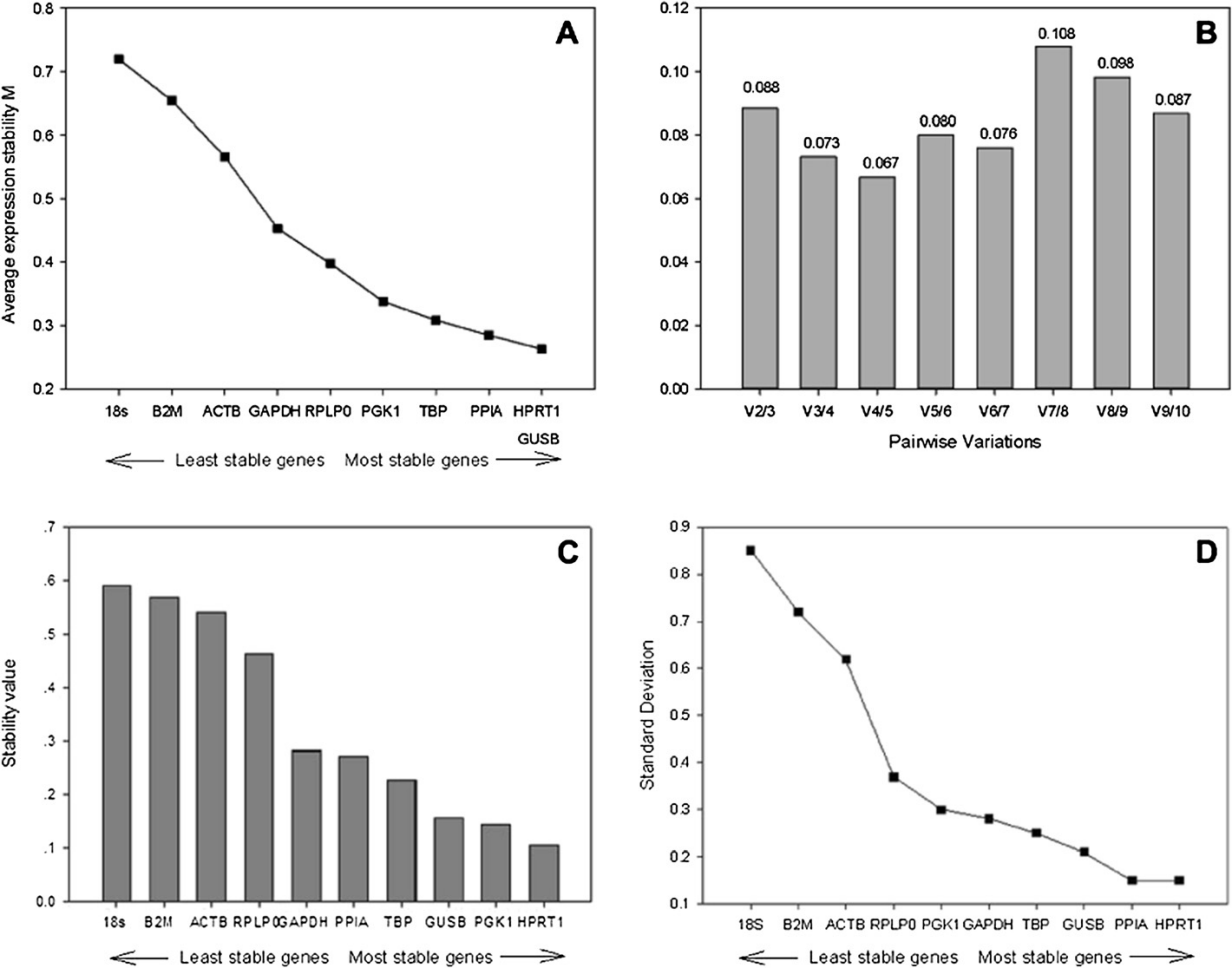
**Stability values of reference gene candidates for hCEC qRT-PCR gene expression studies.** (**A**) Average expression stability values of 10 candidate reference genes by stepwise exclusion of least stable genes, using the comparative Ct methods and analysed using the GeNorm algorithm (**B**) Pair wise variation of candidate gene indicates that 2 reference genes give a low variation so are suitable for normalisation (<0.15), (**C**) Ct values were transformed into relative quantities using the comparative Ct methods and analysed using the NormFinder algorithm (**D**) Standard deviation values calculated using BestKeeper, values <1 indicate a suitable housekeeping gene.

When GeNorm applet was used, *HPRT1* and *GUSB* were recommended as the most stable gene combination with the lowest M values and *18S* was highest (Figure 2A). Pairwise variation analysis of the endogenous genes indicated, in future studies, the sufficiency of two reference genes for accurate normalisation (Figure 2B), there is no need for inclusion of additional reference genes.

NormFinder produced comparable results to GeNorm, *HPRT1* was the most stable slightly more than, *PGK1* and *GUSB* with similar stabilities (Figure 2C). These results confirmed if using a single gene, *HPRT1* has the lowest variability. The least stable reference gene was again *18S* (Figure 2C). Thus, although the underlying principles and calculations of GeNorm and NormFinder are different, there was a correlation between the results obtained from them.

Outputs from the BestKeeper were slightly different from the previous two analyses, as with GeNorm and NormFinder, BestKeeper ranked *HPRT1* as the most stable, but *PPIA* was ranked as the second most stable reference gene, with *GUSB* third (Figure 2D) (Additional file 1). These genes showed the lowest SD according to BestKeeper and were thus considered the most stable reference genes. They were also listed among the three most stable genes by NormFinder and GeNorm, whereas *18S* was consistently ranked the least stable candidate in hCEC by all three programs. However, all of the candidate reference genes could be considered as endogenous genes, since none of their SD values was higher than 1.

### Reference gene validation in hREC

**Figure 3:**
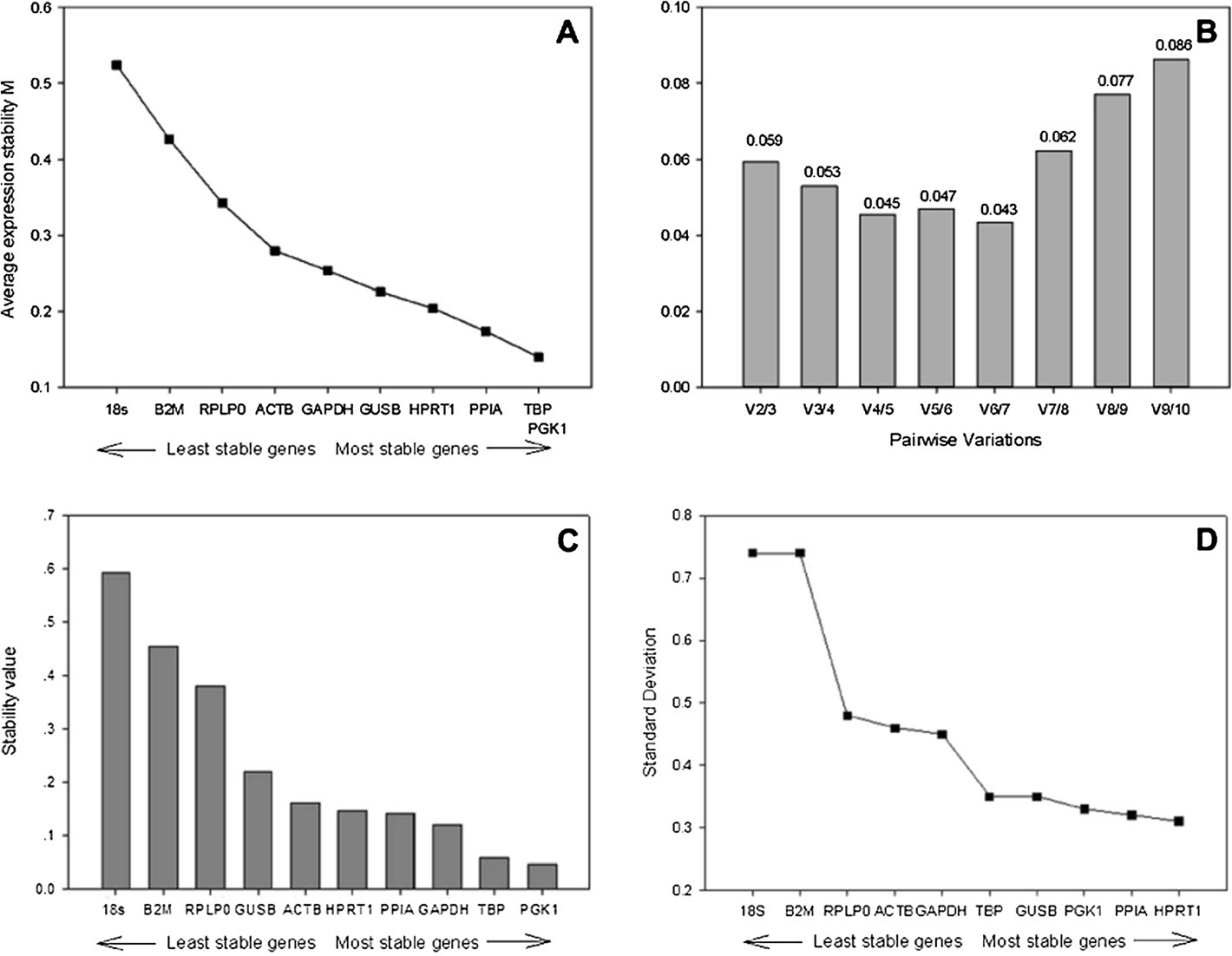
**Stability values of reference gene candidates for hCEC qRT-PCR gene expression studies.** (**A**) Average expression stability values of 10 candidate reference genes by stepwise exclusion of least stable genes, using the comparative Ct methods and analysed using the GeNorm algorithm (**B**) Pair wise variation of candidate gene indicates that 2 reference genes give a low variation so are suitable for normalisation (<0.15), (**C**) Ct values were transformed into relative quantities using the comparative Ct methods and analysed using the NormFinder algorithm (**D**) Standard deviation values calculated using BestKeeper, values <1 indicate a suitable housekeeping gene.

Endogenous gene expression in 12 hREC samples, derived from 3 individual donors (3 unstimulated and 9 stimulated with poly(I:C) for 1h, 6h or 24h) were analysed with each of the 3 mathematical algorithms. Using GeNorm analysis, successive elimination of the least stable genes based on the M values led to the identification of *TBP* and *PGK1* as the most stable pair of reference genes, and 18S was considered as the least stable one in all groups (Figure 3A). These 2 genes with the most stable expression were optimal for reliable normalisation in this study, with a pair wise variation lower than 0.15 (Figure 3B). Therefore, the use of the two most stable genes (*TBP* and *PGK1*) is sufficient for an accurate normalization.

Analysis of the gene expression of candidate references with NormFinder in hREC found *PGK1* as the gene with the lowest stability value (Figure 3C), which was similar to results identified by GeNorm.

Ct values were compared in BestKeeper with various combinations of the candidate genes. All of the candidate reference genes showed a SD value lower than 1 (Figure 3D). Further data processing using Repeated Pair-wise Correlation and Regression Analysis assessed *HPRT1* as the most stable reference gene, with the lowest standard deviation. *18S* had the highest variation and the least correlation.

Manipulation of the different EC with poly(I:C) stimulation did not alter the outputs of the 3 different algorithms. This indicates the ability to reproducibly quantify genes with or without stimulation.

We have identified the most stable endogenous control genes in intraocular endothelial cells. It is suggested that future qPCR studies of human intraocular endothelial cells would benefit from adopting the genes identified in this study as the most appropriate endogenous control genes. *TBP* and *PGK1* together form the best combination of genes for hREC, and *HPRT1* and *GUSB* for hCEC with GeNorm and Norm Finder, whilst BestKeeper ranked *HPRT1* and *PPIA* are the two most stable reference genes for hCEC (Table 2), and *HPRT1* as the most stable reference gene for hREC (Table 3). The least stable gene for hREC and hCEC was *18S* with all 3 algorithms.

**Table 2:** Comparison of ranked endogenous genes in hCEC by all three software

**Gene name**	**GeNorm expression stability (M)**	**NormFinder stability value (ρ)**	**BestKeeper coefficient of correlation (r)**
*18S*	0.979	0.590	0.925
*ACTB*	0.927	0.541	0.668
*B2M*	0.954	0.568	0.658
*GAPDH*	0.676	0.281	0.567
*GUSB*	0.555	0.156	0.479
*HPRT1*	0.531	0.105	0.579
*PGK1*	0.577	0.145	0.722
*PPIA*	0.608	0.270	0.307
*RPLP0*	0.782	0.463	0.485
*TBP*	0.602	0.226	0.408

**Table 3:** Comparison of ranked endogenous genes in hREC by all three software

**Gene name**	**GeNorm expression stability (M)**	**NormFinder stability value (ρ)**	**BestKeeper coefficient of correlation (r)**
*18S*	0.914	0.593	0.826
*ACTB*	0.457	0.161	0.885
*B2M*	0.743	0.453	0.779
*GAPDH*	0.434	0.120	0.922
*GUSB*	0.462	0.219	0.689
*HPRT1*	0.412	0.146	0.830
*PGK1*	0.384	0.045	0.384
*PPIA*	0.412	0.142	0.839
*RPLP0*	0.643	0.379	0.530
*TBP*	0.379	0.059	0.929

## Conclusion

This study provides the validation of reference genes for RT-qPCR in human microvascular endothelial cells. *HPRT1*, *GUSB* and *PPIA* were recommended as the most stable reference genes for hCEC, *HPRT1*, *TBP* and *PGK1* for hREC. The results outlined in this article can be applied for future RT-qPCR studies using these cells, and it also indicates a prerequisite for accurate RT-qPCR expression profiling.

## Methods

This research received approval from the local research ethics committee, Nottingham Q1060301.

### Stimulation of endothelial cells with poly(I:C)

Fresh human posterior segments free of any known ocular disease were obtained from Manchester Eye Bank. hCEC and hREC were isolated and cultured from the unpreserved tissue within 48h of death using a previously described method [27] .After endothelial cell isolation cells were cultured in endothelial growth medium (EGM2-MV, Lonza) and seeded onto fibronectin (Sigma-Aldrich) coated 35mm culture dishes. The cells were incubated at 37°C in a humidified atmosphere of 5% (v/v) CO_2_ and the medium was changed every 2 days. When the cells were nearly 100% confluent, hCEC and hREC from each donor were transferred onto fibronectin (Sigma-Aldrich, Gillingham, UK) coated 6-well plates and grown until they reached 70% confluence. Cells were then incubated with 10μg/mL poly(I:C) (Invivogen, San Diego, USA) for 1h, 6h, and 24h, after which lysates were collected with Buffer RLT (Qiagen, Crawley, UK).

### RNA extraction and cDNA synthesis

Total RNA was extracted and purified from the cells using RNeasy Mini RNA kit (Qiagen) according to the manufacturer’s protocol. The lysate was homogenized by QIAshredder spin column, and centrifuged for 2min at full speed. 1 volume of 70% (v/v) ethanol was added to the homogenized lysate and transferred to RNeasy spin column. Buffer RW1 and Buffer RPE was applied to wash the spin column membrane. Finally, total RNA was eluted with 30μL RNase-free water. All the washing and elution steps mentioned above were followed with stepwise centrifugation. RNA was quantified by using a Nanodrop and the quality was determined by agarose gel electrophoresis.

RNA samples were further processed for reverse transcription using the QuantiTect RT kit (Qiagen). 2μg of the purified RNA was incubated with genomic DNA Wipeout Buffer for 2min at 42°C. The reverse-transcription mastermix, containing QuantiTect RT enzyme, QuantiTect RT Buffer and RT Primer Mix, was prepared and added to the template RNA. Samples were incubated at 42°C for 15min, followed by incubating the samples at 95°C for 3min. A 40μL final volume of cDNA was stored at −20°C for real-time PCR.

### Real-time PCR (RT-PCR)

RT PCR was carried out with Roche Lightcycler 480. All samples were diluted 1:5 with nuclease-free water. A single 20μL reaction mixture was prepared to each PCR optical tube, with 10μL of 10 × Taqman gene expression master mix (Applied Biosystems, Foster City, CA), 1μL of TaqMan® Gene expression assay (Applied Biosystems; Table 4), 5μL of diluted cDNA and 4μL of nuclease-free water, were performed in optical tubes with optical caps on. Negative controls (non-template control and negative reverse transcriptase control) and positive controls (Universal Human Reference RNA) were also run on the PCR. Amplification conditions were set at 50°C for 2min, 95°C for 10min, 95°C for 15s and 60°C for 1min. Each reaction was run in triplicate in a Stratagene MX-3005p Quantitative PCR instrument. A total of 12 samples were analysed from 3 pairs of donor eyes in each treatment group at 3 time points.

**Table 4:** Details of the Taqman gene assays used for qPCR (primer sequences are proprietary)

**Gene name**	**Assay ID**	**Accession number**	**Probe exon location**	**Amplicon size**
*18S*	4352930E	X03205.1	NA	187
*ACTB*	4333762T	NM_001101.2	1	171
*B2M*	4333766T	NM_004048.2	2-3	75
*GAPDH*	4333764T	NM_002046.3	3	122
*GUSB*	4333767T	NM_000181.1	11-12	81
*HPRT1*	4333768T	NM_000194.1	6-7	100
*PGK1*	4333765T	NM_000291.2	4-5	75
*PPIA*	4333763T	NM_021130.3	5	98
*RPLP0*	4333761T	NM_053275.3	3	105

### Data analysis

The gene expression levels for each of 10 selected potential reference genes were calculated and analysed for comparative stability with the three methods: GeNorm, NormFinder and BestKeeper.

## Competing interests

The authors declare that they have no competing interests.

## Authors’ contributions

RW carried out the experimental work involved in the study and drafted the manuscript. ES participated in the design of the study, re-drafted and finalised the manuscript. WMA provided the materials, participated in the design of the study and edited the manuscript. All authors read and approved the final manuscript.

## Supplementary Material

Additional file 1 Results of BestKeeper analyses in untreated and treated hCEC. Click here for file
