# Supplementary material for: Suitability of endogenous reference genes for gene expression studies with human intraocular endothelial cells
Source: BMC Res Notes. 2013 Feb 4;6:46. doi: 10.1186/1756-0500-6-46 (PMC3567942; doi:10.1186/1756-0500-6-46)
Supplement: Additional file 1 — Results of BestKeeper analyses in untreated and treated hCEC. [file 1756-0500-6-46-S1.docx]

**Supplementary Data**

**Table S1** Results of BestKeeper analyses in untreated and treated hCEC

|  | *TBP* | *HPRT1* | *GAPDH* | *GUSB* | *PPIA* | *RPLP0* | *B2M* | *18s* | *PGK1* | *ACTB* |
| --- | --- | --- | --- | --- | --- | --- | --- | --- | --- | --- |
| **GM [CP]** | 26.92 | 24.37 | 19.85 | 22.86 | 20.02 | 19.45 | 18.07 | 13.60 | 23.32 | 18.79 |
| **AM [CP]** | 26.92 | 24.37 | 19.86 | 22.86 | 20.02 | 19.45 | 18.09 | 13.63 | 23.33 | 18.81 |
| **min [CP]** | 26.28 | 23.89 | 18.88 | 22.49 | 19.41 | 18.88 | 16.33 | 12.00 | 22.64 | 17.63 |
| **max [CP]** | 27.82 | 24.86 | 21.40 | 23.49 | 20.43 | 20.34 | 19.39 | 15.46 | 24.27 | 21.58 |
| **SD [±CP]** | 0.25 | 0.15 | 0.28 | 0.21 | 0.15 | 0.37 | 0.72 | 0.85 | 0.30 | 0.62 |
| **CV [%CP]** | 0.94 | 0.62 | 1.42 | 0.90 | 0.73 | 1.88 | 4.00 | 6.26 | 1.27 | 3.32 |

GM, geometric mean; AM, arithmetic mean; Cq, quantification cycle; SD, standard deviation; CV, coefficient of variation.

**Table S2** Results of BestKeeper analyses in untreated and treated hREC

|  | *TBP* | *HPRT1* | *GAPDH* | *GUSB* | *PPIA* | *RPLP0* | *B2M* | *18S* | *PGK1* | *ACTB* |
| --- | --- | --- | --- | --- | --- | --- | --- | --- | --- | --- |
| GM [CP] | 26.69 | 24.41 | 19.87 | 23.23 | 20.56 | 19.85 | 17.96 | 12.80 | 23.31 | 19.32 |
| AM [CP] | 26.69 | 24.41 | 19.88 | 23.23 | 20.57 | 19.85 | 17.98 | 12.84 | 23.31 | 19.33 |
| min [CP] | 25.98 | 23.87 | 19.05 | 22.67 | 19.82 | 18.79 | 16.27 | 11.69 | 22.59 | 18.41 |
| max [CP] | 27.30 | 24.94 | 20.96 | 23.96 | 21.19 | 20.88 | 19.15 | 15.95 | 24.01 | 20.12 |
| SD [±CP] | 0.35 | 0.31 | 0.45 | 0.35 | 0.32 | 0.48 | 0.74 | 0.74 | 0.33 | 0.46 |
| CV [%CP] | 1.31 | 1.28 | 2.27 | 1.51 | 1.58 | 2.42 | 4.12 | 5.78 | 1.41 | 2.37 |

GM, geometric mean; AM, arithmetic mean; Cq, quantification cycle; SD, standard deviation; CV, coefficient of variation;
